# Influence of glutamine on transient and stable recombinant protein production in CHO and HEK-293 cells

**DOI:** 10.1186/1753-6561-5-S8-P35

**Published:** 2011-11-22

**Authors:** Yashas Rajendra, Divor Kiseljak, Lucia Baldi, David L Hacker, Florian M Wurm

**Affiliations:** 1 Laboratory for Cellular Biotechnology (LBTC), École Polytechnique Fédéral de Lausanne (EPFL), CH-1015 Lausanne, Switzerland

## Background

Glutamine is an essential component in culture media for most of the mammalian cell lines. It is often used as an alternative source of energy by cells, along with glucose. Glutamine metabolism induces ammonia accumulation in cell culture. Elevated ammonia concentration above 2 mM has been shown to have negative impact on both cell growth and recombinant protein productivity [1-4]. In this study we investigated the effects of decreased glutamine concentration in the medium for CHO-DG44 and HEK-293E cells during transient gene expression (TGE). The rationale was to reduce ammonia accumulation in the culture, and consequently, improve cell viability and recombinant protein productivity.

## Materials and methods

### CHO-DG44 transfection

The cells were centrifuged and resuspended in ProCHO5 medium (Lonza, Verviers, Belgium) at a density of 5x10^6^ cells/mL in orbitally shaken 250 ml glass bottles. Each transfection was performed in 100 mL of culture using 0.6 µg of plasmid DNA and 3.0 µg of linear 25 kDa polyethyleneimine (PEI, Polysciences, Eppelheim, Germany; pH 7) per million cells. The transfected cultures were incubated at 31 °C in 5% CO2 and 85% humidity with agitation at 120 rpm.

### HEK-293E transfection

The cells were centrifuged and resuspended at density of 20x10^6^ cells/mL in RPMI 1640 medium. Each transfection was performed in 100 mL of culture using 1.5 µg of plasmid DNA and 3.0 µg of linear 25 kDa PEI per 10^6^ cells. Three hours post-transfection, cells were diluted with Ex-Cell293 medium (Sigma, Saint-Louis, USA) to a density of 2x10^6^ cells/mL, and valproic acid was added to a final concentration of 3.75 mM. The transfected cultures were incubated at 37 °C in 5% CO_2_ and 85% humidity with agitation at 120 rpm.

### Stable Clone and Pool

A cell line and cell pool expressing anti-Rhesus IgG was kindly provided by Tatiana Benavides from our lab. Cell line was established by transfection of plasmid containing both light and heavy chain into CHO-DG44 cells, followed by flow cytometer sorting and limiting dilution. Cell pool was established by transfection of plasmid containing both light and heavy chain into CHO-DG44 cells, followed by selection under puromycin for two weeks.

### Metabolic analytes and IgG levels

The levels of glucose, glutamine, ammonium, and lactate were determined with a BioProfile 200 Bioanalyzer (Nova Biomedical Corp., Waltham, MA). The IgG concentration in the culture medium was determined by sandwich ELISA as previously described [5].

## Results

### Low glutamine concentration improved transient IgG production

**Figure 1:**
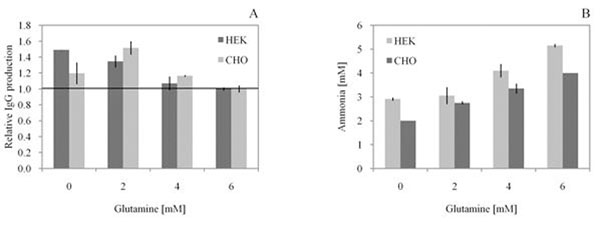
Effect of glutamine concentration on relative A) IgG production and B) Ammonia accumulation in CHO-DG44 cells and HEK-293E cells under transient transfection conditions on day 7.

Different concentrations of free glutamine in medium, ranging from 0 mM to 6 mM were tested. Both cell lines showed improved production of IgG with a reduced glutamine concentration (Fig. 1 panel A). The optimal concentration of glutamine in terms of IgG production was 2 mM for CHO-DG44 cells and 0 mM for HEK-293E cells. We observed a 50% improvement in IgG production for both CHO-DG44 and HEK293 cells. We did not observe a significant difference on cell density or viability between the tested concentrations of glutamine for both CHO-DG44 and HEK293 cells during the entire course of the culture (data not shown).

Higher initial concentration of glutamine resulted in higher concentration of ammonia, up to 5 mM for the highest glutamine concentration tested (Fig 1 panel B).

Lactate accumulation in both CHO-DG44 and HEK-293E cells was observed during the initial phase of the cultures, while the cells were actively growing. In CHO-DG44 cells, consumption of lactate started immediately after its accumulation. In HEK-293E cells, lactate levels remained either at a constant level or, in the absence of glutamine, continued to increase over the entire culture (data not shown). Glucose and glutamine consumption rate remained unaffected under all the conditions tested.

### Lower initial glutamine concentration improved stable IgG production in growth-arrested stable CHO-DG44 cells and pools

A stable cell clone and cell pool of CHO-DG44 cells expressing IgG were cultivated in the presence of different glutamine concentrations under mild hypothermia conditions (31 °C). Both the clone and the cell pool showed improved production of IgG with reduction in glutamine concentration. The IgG titers were approximately 80% higher for the clone and 60% higher for the pool at 0 mM glutamine, compared to titers obtained with 6 mM glutamine (data not shown).

## Conclusions

The effects of different concentrations of glutamine on IgG production in growth arrested cells were investigated. Recently published results show that CHO-DG44 cells are arrested in G1 phase of the cell cycle under mild hypothermia at 31 °C [6]. For HEK-293E cells growth arrest was induced with VPA [7]. We conclude that a lower glutamine concentration results in improved transient antibody titers in CHO-DG44 and HEK-293E cells mainly due to lower accumulation of ammonia in the culture, which has previously been shown to have a negative impact on cellular productivity [1-4]. Glutamine reduction also had a positive impact on recombinant IgG production in a stable clone and a pool of recombinant CHO-DG44 cells at 31°C. These data suggest that this strategy may be successful in both transient and stable gene expression processes under conditions of growth arrest.
